# Antiviral Agents for the Prevention and Treatment of Herpes Simplex Virus Type-1 Infection in Clinical Oncology: A Network Meta-Analysis

**DOI:** 10.3390/ijerph17238891

**Published:** 2020-11-30

**Authors:** Farah Wasim Aribi Al-Zoobaee, Loo Yee Shen, Sajesh K. Veettil, Divya Gopinath, Mari Kannan Maharajan, Rohit Kunnath Menon

**Affiliations:** 1 School of Postgraduate Studies, International Medical University, Kuala Lumpur 57000, Malaysia; farah.wasim@student.imu.edu.my; 2 School of Pharmacy, International Medical University, Kuala Lumpur 57000, Malaysia; loo.yeeshen@student.imu.edu.my; 3 Department of Pharmacotherapy, College of Pharmacy, University of Utah, Salt Lake City, UT 84132, USA; sajesh.vijay@gmail.com; 4 Oral Diagnostics and Surgical Sciences, School of Dentistry, International Medical University, Kuala Lumpur 57000, Malaysia; divyagopinath@imu.edu.my; 5 Department of Pharmacy Practice, School of Pharmacy, International Medical University, Kuala Lumpur, 57000, Malaysia; marikannan@imu.edu.my; 6 Division of Clinical Dentistry, School of Dentistry, International Medical University, Kuala Lumpur 57000, Malaysia

**Keywords:** herpes simplex virus, cancer, antiviral, prophylaxis, systematic review, network meta-analysis

## Abstract

Cancer therapy may be complicated and compromised by viral infections, including oral herpes simplex virus (HSV) infection. This network meta-analysis aimed to identify the best antiviral agent to prevent or treat oral HSV infection in patients being treated for cancer. A search was conducted for trials published since inception until the 10th of May 2020 in MEDLINE, EMBASE and the Cochrane Central Register of Controlled Trials. A network meta-analysis was performed on the data from randomized controlled trials that assessed antiviral agents for preventive or therapeutic activity vs. placebo, no treatment or any other active intervention in patients being treated for cancer. The agents were ranked according to their effectiveness in the prevention of oral HSV using surface under the cumulative ranking (SUCRA). Grading of Recommendations, Assessment, Development and Evaluations (GRADE) was used to assess the certainty of the evidence. In total, 16 articles were included. The pooled relative risk (RR) to develop oral HSV infection in the acyclovir group was 0.17 (95% CI: 0.10, 0.30), compared to 0.22 (95% CI: 0.06, 0.77) in the valacyclovir group. Acyclovir ranked highest for the prevention of oral HSV followed by valacyclovir. Subgroup analysis with different acyclovir regimens revealed that the best regimens in terms of HSV-1 prevention were 750 mg/m^2^ acyclovir administered intravenously followed by 1600 mg per day orally. Acyclovir (250 mg/m^2^ per day) administered intravenously was the least effective against the prevention of oral HSV.

## 1. Introduction

Cancers are currently the second major cause of death worldwide after cardiovascular disease [1]. Treatment of cancer is usually associated with many complications that may generate additional burden affecting the patient’s quality of life and could cause pain, difficulty in swallowing, mucositis, salivary gland dysfunction, malnutrition, anemia, weight loss, and can potentially cause sepsis leading to life-threatening systemic infections [2]. Oral mucositis is one of the most common and troublesome adverse effect of many therapeutic strategies of cancer (chemotherapy, radiotherapy, and chemo-radiotherapy), which can produce pain, ulceration, malnutrition, and may impact the treatment as well [3]. Impact on the treatment regimen can be considered as a serious adverse effect which may be the consequence of an oral infection. Oral infections could be attributed to bacterial, fungal, or viral infections, which are leading causes of morbidity and mortality in patients undergoing cancer treatment.

Herpes Simplex Virus (HSV) is a very common viral infection and the majority of the people are usually infected early in their life. The prevalence of HSV differs according to age, socioeconomic status, and geographic location [4,5,6].

HSV can cause several types of diseases, ranging from labialis and stomatitis to blinding keratitis and, rarely, encephalitis. The clinical symptoms accompanying oral HSV ranges from asymptomatic infection to life-threatening diseases. Gingival stomatitis and pharyngitis are the most common manifestations of the first episode. Symptoms such as fever, chills, fatigue, muscle aches, and blisters on or around the lips and within the mouth will be experienced by the patient and will last for 10–14 days. The incubation period of HSV-1 ranges from 1 to 26 days, with a median range of 6 to 8 days [7,8,9]. After the primary infection, the virus tends to reside in the trigeminal ganglia; this persistent form is known as a latent infection. However, HSV-1 can episodically reactivate from latency, and it is estimated that one-fourth of people who have seropositive HSV test will develop oral HSV reactivation once or four times per year [9,10,11]. Recurrence mainly occurs after the virus gets triggered by a stimulus [12]. Triggers include sunlight, menstruation, stress, trauma, febrile illness, chemotherapy, radiotherapy, or immunosuppression [13,14]. Dreizen et al. [15], reported that in 1000 patients receiving cancer treatment, 9.7% of them were diagnosed with an oral infection, and of these, 10.7% were due to viruses. In previous studies, the prevalence of oral infection in immunocompromised participants is under-reported, and up to 40–50% of these patients were reported to be diagnosed with herpetic infections [16,17,18]. Oral HSV infection is known to be more aggressive in patients being treated for cancer when compared to immunocompetent individuals. Reactivated HSV-1 infections develop as large intraoral ulcers or extensive necrotic lesions around the mouth. HSV-1 esophagitis rarely develops in immunocompetent subjects; however, it is usually seen in immunocompromised individuals. Up to 80% of seropositive HSV patients receiving allogeneic stem cell transplants have been reported to develop HSV-1 reactivation episodes [19]. More than 60% of patients with acute leukemia receiving chemotherapy have been previously reported to develop HSV-1 reactivation [20,21,22,23]. Antiviral prophylaxis has been recommended for HSV-seropositive patients with leukemia as per the guidelines of the Second European Conference on infections in leukemia [24]. Further, several guidelines have recommended antiviral prophylaxis for patients undergoing haematopoietic stem cell transplantation against HSV-1 [25,26,27].

Owing to this morbidity, there has been much effort in developing antiviral agents to treat and prevent oral HSV in patients undergoing cancer treatment. The main aims of antiviral drugs for HSV encompass hindrance of HSV reactivation, pain reduction, reducing the duration of viral shedding and symptoms [28]. Several types of antiviral agents such as acyclovir, famciclovir, penciclovir, and valacyclovir have been recommended for the treatment and/or prevention of HSV-1 [29]. All these agents are available as oral dosage forms, but only acyclovir is available as a parenteral and topical formulation. Acyclovir requires more frequent dosing due to its poor bioavailability which is estimated to be only 20%, and its plasma half-life which is approximately 3 h [30]. At the same time, valacyclovir and famciclovir have good oral bioavailability, thus requiring less frequent dosing. All drugs used for the treatment or prevention of HSV have a wide safety margin because they will be activated by the viral thymidine kinase only inside the infected cells [31].

Evidence regarding the effectiveness of antiviral agents in the prevention or treatment of oral HSV in patients undergoing cancer treatment is still inconclusive. Hence, this systematic review and meta-analysis aimed to enhance the understanding of the oral HSV infection burden and complications in patients receiving cancer regimens and highlights the importance of using the most effective and safe antiviral agent to treat and prevent oral HSV infection.

## 2. Materials and Methods

### 2.1. Search Strategy

This systematic review and network meta-analysis (NMA) was conducted based on the general principles outlined in the Cochrane Handbook for Systematic Reviews of Interventions, and it was reported based on the Preferred Reporting Items for Systematic Reviews and Meta-Analyses (PRISMA) extension statement for network meta-analysis [32,33]. An inclusive search of randomized controlled trials published from inception until the 10th of May 2020 was conducted in Medline, Cochrane Central Register of Controlled Trials and Embase. The search strategies and search terms used in Embase, Medline, and Cochrane Central Register of Controlled Trials are documented in Table A1. in Appendix A. The search was restricted to publications in the English language and human studies. For additional studies, references of previously published reviews were scanned.

Selection criteria based on the Participants (Ps), Intervention (I), Comparisons (Cs) and Outcomes (Os) statements are given below:

- Participants: Patients undergoing cancer treatment. Adults, children, or both were included in the search.
- Intervention: Any antiviral agent used for the prevention or treatment of oral herpes simplex virus infection were included.
- Comparisons: Active intervention, placebo, or no treatment.
- Outcomes: The primary outcome analyzed for prevention was the occurrence or absence of herpes simplex virus infections, clinically or as a positive culture. The primary outcome analyzed for treatment was the time required for complete healing of the lesions. Secondary outcomes were recurrence of lesions, the time required for pain relief, duration of viral shedding, and adverse effects.

### 2.2. Data Extraction and Management

Titles and abstracts of citations from the electronic databases were screened for relevant articles and the full-text of all potentially relevant articles were extracted. The inclusion and exclusion criteria were applied for all potentially eligible articles. When studies had duplicate publications with the same data, the most recent relevant data from these publications were included in the review and analysis. Two reviewers conducted the screening and selection process and a consensus was obtained; in case of any disagreement, a third reviewer was consulted. The extraction form was created following the Cochrane Handbook for Systematic Reviews of Interventions guidelines by consensus amongst all the reviewers. The data from randomized controlled trials (RCTs) were extracted and separated into the following sections: study characteristics, population characteristics, intervention characteristics, and outcome definitions and measures. For all outcomes, the initial number of participants randomized to each trial arm was used. We performed the analyses irrespective of how the authors of the original trials had analyzed the data (intention-to-treat principle) [34].

### 2.3. Quality of Assessment

Cochrane risk of bias tool 2.0 was used to evaluate the risk of bias of each study [35].

### 2.4. Strategy for Data Synthesis

Data were analyzed based on the intention-to-treat principle. Risk ratios and 95% confidence intervals were used as summary statistics in this network meta-analysis. A standard pairwise meta-analysis was performed by using a random-effects (DerSimonian and Laird) model for direct comparisons [32]. If a direct comparison was based on two or more studies, heterogeneity between the trials was evaluated by considering the I2 statistics; an I2 estimate ≥50% was interpreted as evidence of substantial levels of heterogeneity [32]. A random-effects network meta-analysis was applied to synthesize the available evidence by combining direct and indirect evidence from different studies [36,37]. Network inconsistency, which refers to a disagreement between direct and indirect estimates, was assessed using a global inconsistency test by fitting a design-by-treatment model. A network plot was used to map different interventions and placebo.

## 3. Results

### 3.1. Study Selection

**Figure 1:**
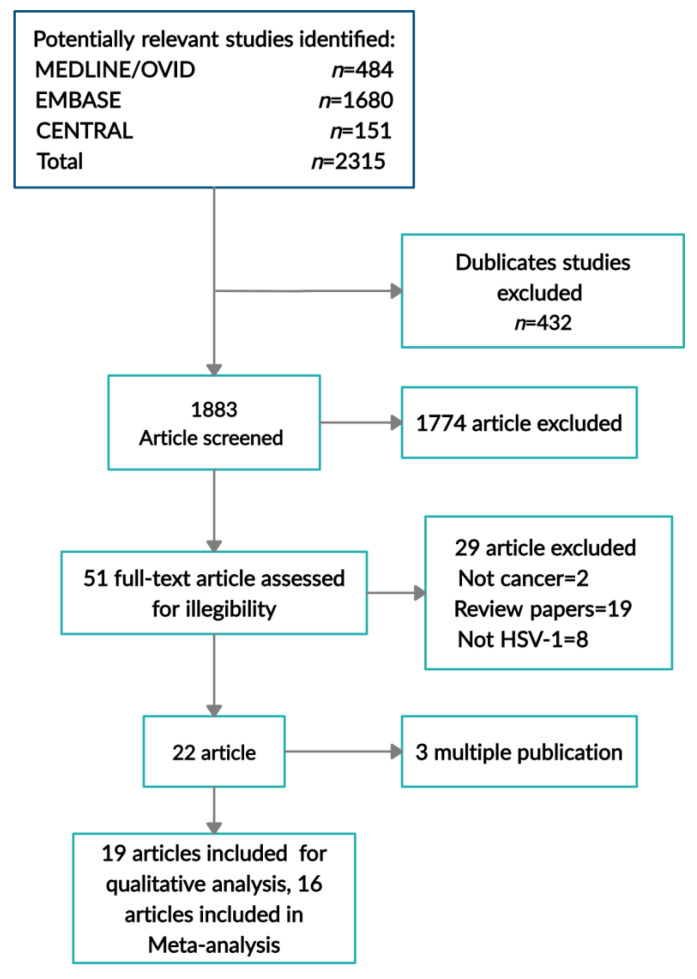
Preferred Reporting Items for Systematic Reviews and Meta-Analyses (PRISMA) flow diagram summarizing the study selection process for eligible trials.

The search yielded 2315 potentially relevant publications (151 Central, 1680 Embase, and 484 Medline). A total of 432 articles were eliminated due to duplication, following which 1883 articles underwent screening based on the title and abstract. A total of 51 full-text articles were reviewed and three articles which presented the same data were eliminated due to multiple publications [38,39,40]. Eventually, 19 randomized controlled trials [20,41,42,43,44,45,46,47,48,49,50,51,52,53,54,55,56,57,58] which met the inclusion criteria were included in the qualitative review and 16 [20,41,42,43,44,45,46,47,48,49,50,51,52,53,54,55] articles were included in the meta-analysis. Figure 1 depicts the trial selection flow chart.

### 3.2. Study Characteristics

**Figure 2:**
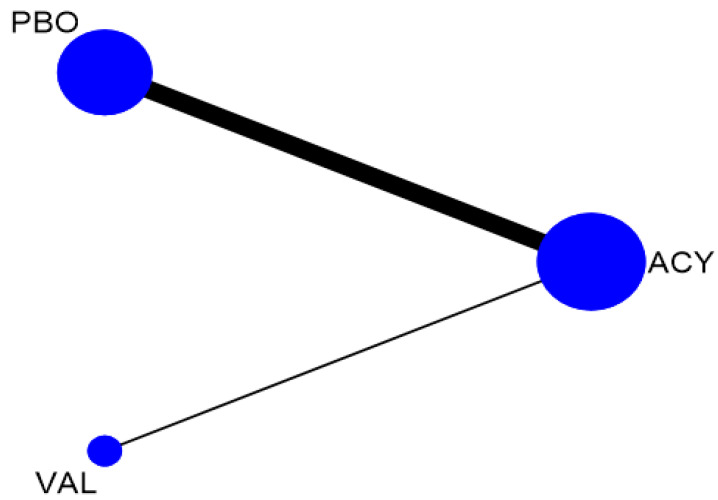
Network plot of herpes simplex virus incidence by drug class. Notes: The size of the node and the thickness of the straight line are proportional to the number of trials and the presence of direct evidence between compared agents. Abbreviations: PBO, placebo; ACY, acyclovir; VAL, valacyclovir.

Table 1 shows the characteristics of the included RCTs. Trials included were published from 1981 to 2002. The drugs assessed included acyclovir in oral and intravenous (IV) dosage forms and valacyclovir. Among the 19 trials, a total of 16 studies with 949 participants comparing three interventions were included in the NMA for the efficacy of antifungal agent in preventing oral HSV infection in patients undergoing cancer treatment (Figure 2) [20,41,42,43,44,45,46,47,48,49,50,51,52,53,54,55]. These included 14 trials comparing acyclovir with placebo [20,41,42,43,44,45,46,47,48,49,50,51,52,53] while another two trials compared valacyclovir and acyclovir [54,55]. The study by Orlowski (2004) was excluded from this analysis because the study was comparing two different valacyclovir doses (500 vs. 1000 mg) and hence cannot be connected to the network loop [56]. For the treatment outcome, only two trials assessed acyclovir activity [57,58] whereas none of the available trials evaluated the efficacy of valacyclovir.

#### Quality of RCTs

Quality assessment of each study using the Risk of Bias (ROB) assessment tool is provided in Table 2. Among the RCTs, 10 trials were evaluated and found to have a high ROB, whereas the remaining studies were at low or some concern ROB.

Based on the NMA results, acyclovir (RR, 0.17 (95% CI: 0.10, 0.30)) and valacyclovir (RR, 0.22 (95% CI: 0.06, 0.77)) significantly reduce the risk of developing oral HSV infection when compared to placebo. No significant difference was observed for the comparison on efficacy between acyclovir and valacyclovir in the prevention of oral HSV (RR, 0.79, (95% CI: 0.25, 2.48)) (Table 3).

**Figure 3:**
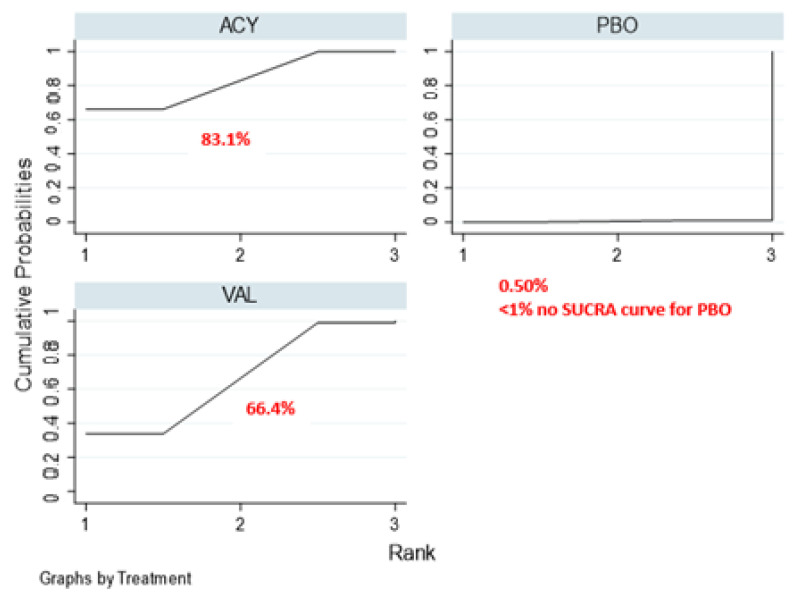
SUCRA ranking curve of antiviral agents for the prevention of oral herpes simplex virus infection. Abbreviations: ACY, acyclovir; VAL, valacyclovir; PBO, placebo.

Surface under the cumulative ranking (SUCRA) ranks and curves for efficacy outcomes are presented in Table 4 and Figure 3. Acyclovir was ranked the best in preventing oral HSV infection (SUCRA score—0.83), followed by valacyclovir (SUCRA score—0.66) and placebo.

**Figure 4:**
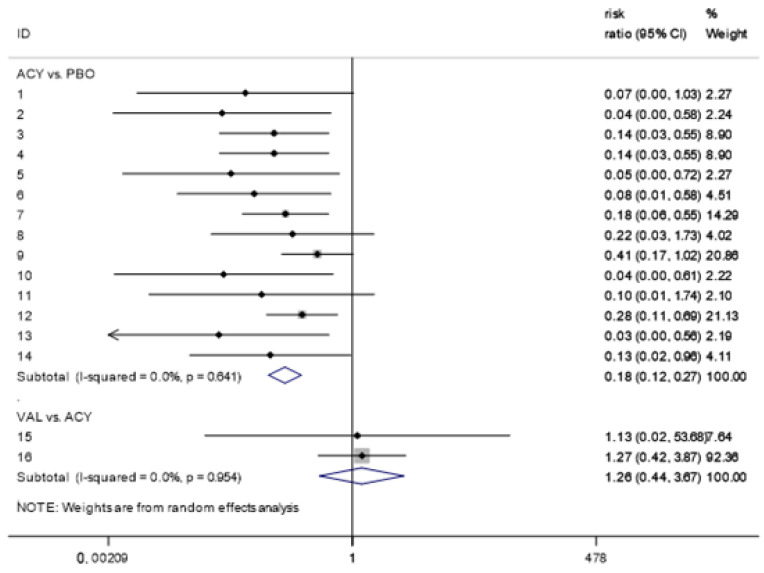
Forest plot of pairwise meta-analysis analyzing the antiviral prophylaxis efficacy against oral HSV. Note: 1, Saral-1981; 2, Gluckman-1983; 3, Hann-1983; 4, Prentice-1983; 5, Saral-1983; 6, Anderson-1984; 7, Wade-1984; 8, Lundgren-1985; 9, Shepp-1985; 10, Shepp-1987; 11, Bubley-1989; 12, Selby-1989; 13, Lonnqvist-1993; 14, Bergmann-1995; 15, Liesveld-2002; 16, Warkentin-2002.

According to the pairwise meta-analysis (Figure 4), acyclovir causes a significant reduction in the risk of oral HSV when compared to placebo (RR, 0.18 (95% CI: 0.12, 0.27)). No significant difference was observed for the comparisons between valacyclovir and acyclovir (RR, 1.26 (95% CI: 0.44, 3.67)). Heterogeneity was low for the included studies between acyclovir vs. placebo and valacyclovir.

Subgroup analysis: comparison of the total daily dose of acyclovir and dosage forms in the prevention of oral HSV incidence.

**Figure 5:**
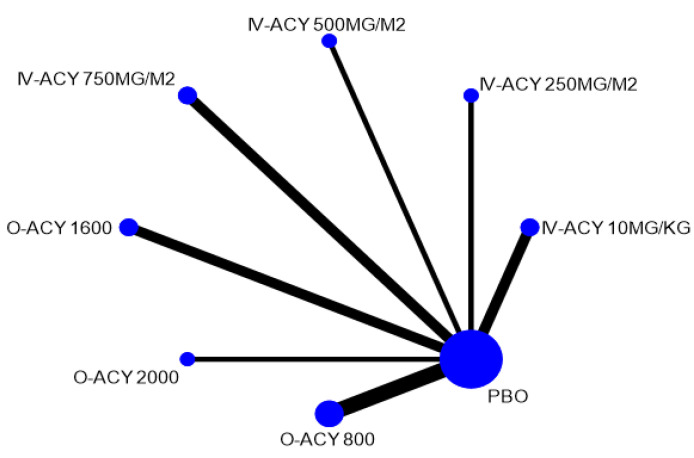
Network plot: incidence of herpes simplex virus with different acyclovir regimens. Abbreviations: PBO, placebo; O-ACY, oral acyclovir; IV-ACY, intravenous acyclovir.

A total of 13 trials comparing eight interventions were included in the subgroup analysis to assess the efficacy of different acyclovir doses in the prevention of oral HSV incidence in patients undergoing cancer treatment (Figure 5) [20,41,42,43,44,45,46,47,48,49,50,52,53]. The study by Selby (1989) [51] was eliminated from the analysis since the study included both children and adults in the treatment group with two different doses—the total daily dose for adults was 3200 mg oral acyclovir, whereas 1600 mg oral acyclovir was given to children. However, the overall result of this study mixed children and adults together.

**Figure 6:**
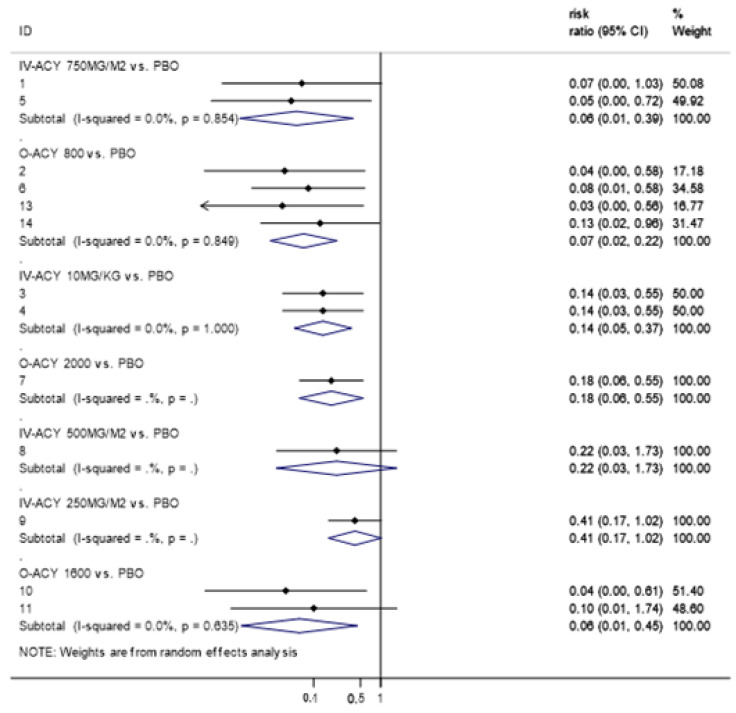
Forest plot of pairwise meta-analysis for studies assessing the efficacy of different acyclovir regimens in preventing oral HSV in patients being treated for cancer. Note: 1, Saral (1981); 2, Gluckman (1983); 3, Hann (1983); 4, Prentice (1983); 5, Saral (1983); 6, Anderson (1984); 7, Wade (1984); 8, Lundgren (1985); 9, Shepp (1985); 10, Shepp (1987); 11, Bubley (1989); 13, Lonnqvist (1993); 14, Bergmann (1995). Abbreviations: PBO, placebo; O-ACY, oral acyclovir; IV-ACY, intravenous acyclovir.

Pairwise meta-analysis was conducted for the seven different acyclovir regimens based on dosing (Figure 6). Most acyclovir regimens were significantly better than placebo at preventing oral HSV infection except for intravenous acyclovir at 500 and 250 mg/m^2^. Compared to placebo, oral acyclovir at 800 mg per day (RR, 0.07 (95% CI: 0.02–0.22)), oral acyclovir at 1600 mg per day (RR, 0.06 (95% CI: 0.01–0.45)), intravenous acyclovir at 10 mg/kg per day (RR, 0.14 (95% CI: 0.05–0.37)), intravenous acyclovir at 750 mg/m^2^ (RR, 0.06 (95% CI:0.01–0.39)) and oral acyclovir at 2000 mg (RR, 0.18 (95% CI: 0.06–0.55)) significantly reduced the development of oral HSV infection. Overall, the statistical heterogeneity was low (I^2^ = 0.0%).

Based on the NMA (Table 5), compared to placebo, all the examined dose were statistically significant in terms of reducing the incidence of oral HSV infection except for the intravenous doses of 500 and 250 mg/m^2^. Intravenous acyclovir (750 mg/m^2^) (RR, 0.06 (95% CI: 0.01, 0.39)) and intravenous acyclovir (10 mg/kg) (RR, 0.14 (95% CI: 0.05, 0.37)) significantly reduced the risk of oral HSV. Among oral acyclovir, the total daily dose of 800 (RR, 0.07 (95% CI: 0.02, 0.22)), 1600 (RR, 0.06 (95% CI: 0.01, 0.45)) and 2000 mg (RR, 0.18 (95% CI: 0.06, 0.55)) was associated with a significant reduction in the incidence of oral HSV infection.

**Figure 7:**
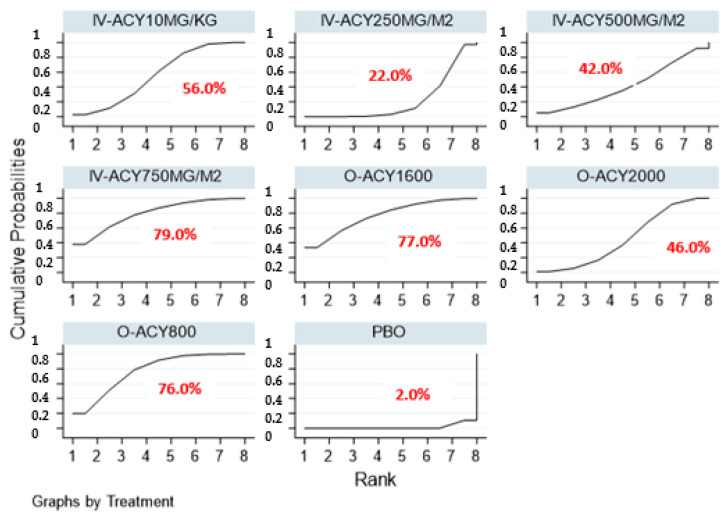
SUCRA ranking curve of different acyclovir regimens. Abbreviations: PBO, placebo; O-ACY, oral acyclovir; IV-ACY, intravenous acyclovir. Network consistency and small study effects. No inconsistency was observed for any outcome in the network meta-analysis (NMA). (Table 7).

When comparing the efficacy between different total daily doses and dosage forms of acyclovir, there was no significant reduction observed in the incidence of oral HSV infections (Table 5). SUCRA ranks and curves for the efficacy of different acyclovir regimens in the prevention of oral HSV infection in patients receiving cancer treatment are provided in Table 6 and Figure 7. Among the interventions, 750 mg/m^2^ intravenous acyclovir (SUCRA score—0.79) was ranked the best in the prophylaxis of oral HSV infection, followed by 1600 mg oral acyclovir (SUCRA score—0.77), 800 mg oral acyclovir (SUCRA score—0.76), 10 mg/kg intravenous acyclovir (SUCRA score—0.56) and 2000 mg oral acyclovir (SUCRA score—0.46).

Pairwise (upper right portion) and network (lower left portion) meta-analytic results. Outcomes are expressed as risk ratios (95% confidence intervals). For the pairwise meta-analyses, a relative risk less than 1 indicates that the treatment specified in the row is more efficacious. For the network meta-analysis, a relative risk less than 1 indicates that the treatment specified in the column is more efficacious. Bold and shaded results indicate statistical significance. Abbreviations: PBO, placebo; O-ACY, oral acyclovir; IV-ACY, intravenous acyclovir.

**Figure 8:**
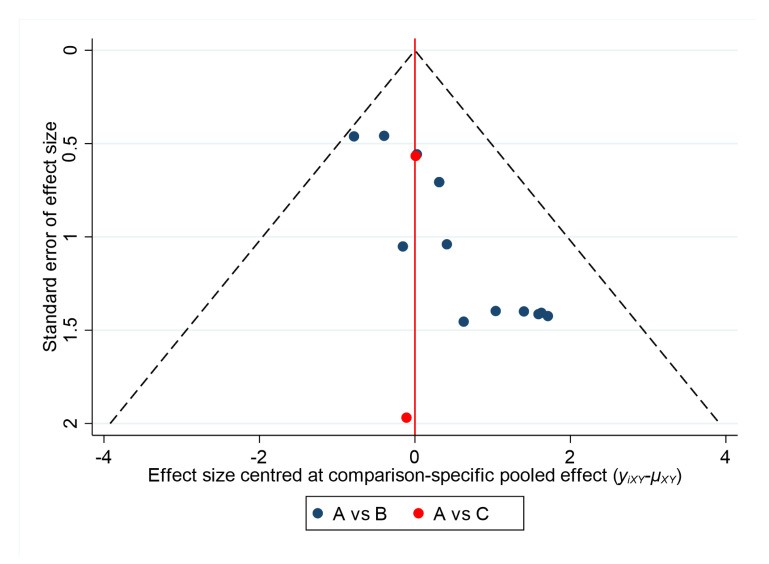
Comparison-adjusted funnel plot for incidence of HSV by drug class. Abbreviations: A = acyclovir; B = placebo; C = valacyclovir.

The comparison-adjusted funnel plot for the incidence of HSV by drug class (Figure 8) was asymmetrical, suggesting that the results might be influenced by the sample size of the literature and publication bias.

**Figure 9:**
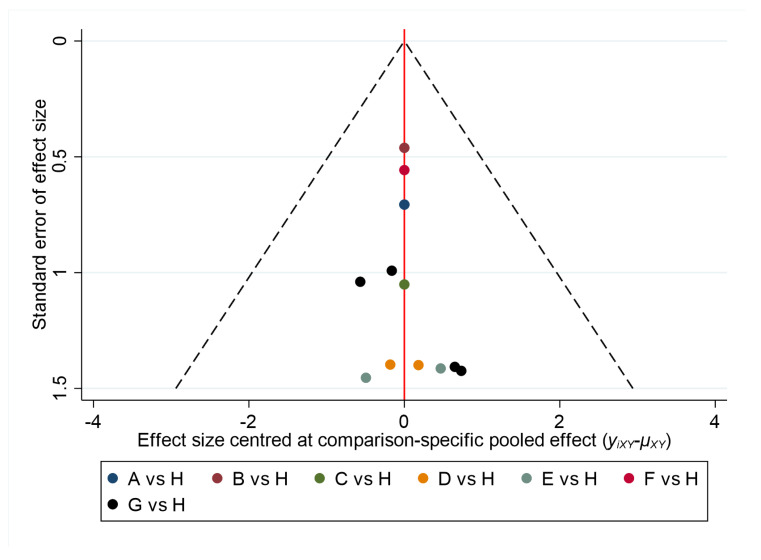
Comparison-adjusted funnel plot for incidence of HSV for different dosage forms and total daily doses. Abbreviations: A, IV acyclovir 10 mg/kg; B, IV acyclovir 250 mg/m^2^; c, IV acyclovir 500 mg/m^2^; D, IV acyclovir 750 mg/m^2^; E, oral acyclovir 1600 mg per day; F, oral acyclovir 2000 mg per day; G, oral acyclovir 800 mg per day; H, placebo.

Based on the comparison-adjusted plots for different dosage forms and total daily doses (Figure 9), no substantial evidence of a small study effect was observed for any outcomes.

### 3.3. Quality of Evidence

The detailed information on the GRADE summary of direct, indirect and NMA evidence for the prevention of oral HSV infection in patients undergoing cancer treatment are presented in Table 8 and Table 9, respectively. GRADE evidence of NMA for the efficacy outcome in the primary and subgroup analysis was rated from moderate to high.

### 3.4. Prevention of Oral HSV Infection Using Valacyclovir

Due to the limited number of trials evaluating different valacyclovir doses in the prevention of oral HSV infection in patients treated for cancer, a network meta-analysis could not be performed. There were only three RCTs evaluating the efficacy of valacyclovir with the total daily doses ranging from 500 to 2000 mg in preventing oral HSV infection [54,55,56]. Based on the results of three RCTs, they indicated that valacyclovir was effective in preventing this oral infection among cancer patients as there is no incidence of HSV infection in the valacyclovir-treated group.

### 3.5. Treatment of Oral HSV Infection

Due to the small number of studies assessing the activity of antiviral agents in the treatment of oral HSV in patient being treated for cancer, NMA could not be performed.

### 3.6. Secondary Outcomes

NMA was not performed for the secondary outcomes due to the limited number of studies.

## 4. Discussion

Defining appropriate therapeutic strategies for secondary infections in cancer is challenging due to the multiple levels of immunodeficiency experienced by the patients. Prevention has always remained one of the important strategies to achieve the aforementioned goal.

Using data from randomized control trials, a network meta-analysis was performed comparing the efficacy of antiviral agents’ in the prevention and treatment of oral HSV infection in patients treated for cancer. To our knowledge, this is the first network meta-analysis that has ranked antiviral agents according to their activity as protective agents against oral HSV infection recurrence, as well as ranking the effectiveness of different acyclovir regimens in the prevention of oral HSV for patients receiving cancer treatment.

Specifically, the results of our network meta-analysis suggest that acyclovir can significantly reduce the relative risk of developing oral HSV infection in patients undergoing cancer treatment. Similarly, valacyclovir shows a significant decrease in the incidence of oral HSV infection in patients undergoing cancer therapy. Our analysis included 16 different RCTs and, according to GRADE, the overall quality of evidence for the network meta-analysis was moderate. Ranking of interventions is one of the most appealing elements of the network meta-analysis. Our network meta-analysis suggests that, in comparison with acyclovir and valacyclovir, acyclovir ranks highest for reducing oral HSV infection incidents in patients being treated for cancer. The visual appearance of the comparison-adjusted funnel plot was asymmetrical, which suggests the existence of publication bias. With respect to the pairwise meta-analysis, the overall pooled relative risk of the acyclovir arm was significantly low, whereas the valacyclovir result was statistically insignificant.

Thirteen RCTs assessing different acyclovir regimens were used to compare the effectiveness of different acyclovir regimens in the prevention of oral HSV infection in patients undergoing cancer treatment. Acyclovir is prescribed in various dosages in an IV form as well as oral formulation. Our network meta-analysis suggests that among the IV formulations, only 750 mg/m^2^ and 10 mg/kg IV regimens were effective in the prevention of oral HSV infections. On the contrary, a total oral daily dose of acyclovir at 800, 1600 as well 2000 mg was effective in the protection against oral HSV infection recurrence. IV acyclovir (750 mg/m^2^), oral acyclovir (1600 mg per day) and oral acyclovir (800 mg per day) were the best regimens in terms of oral HSV infection prevention in patients receiving cancer treatment. On the other hand, 250 mg/m^2^ per day IV acyclovir was the least effective against the prevention of oral HSV infection recurrence in the same population. As seen from our network meta-analysis results, since acyclovir at 800 mg per day has an almost similar activity to acyclovir at 1600 mg per day with a SUCRA score of 76 and 77%, respectively, clinicians might consider prescribing acyclovir at 800 mg per day as the first line in children and geriatric populations and especially patients with renal impairment. According to H. Izzedine et al. [59], higher doses and prolonged durations of nephrotoxic drugs such as acyclovir increase the risk of acute kidney injury in cancer patients.

According to GRADE, the overall quality of evidence for the subgroup network meta-analysis was high-moderate. However, publication bias was not observed in a comparison-adjusted funnel plot which highlighted the robustness of the selected studies.

Comparing the results of pairwise and network meta-analyses, the prophylactic activity of acyclovir was similar in both methods; however, the network meta-analysis results indicate that valacyclovir is an effective agent in the prevention of oral HSV infection reactivation, while the pairwise meta-analysis favored placebo and valacyclovir results were statistically insignificant.

Valacyclovir can be considered as an effective agent in the prevention of oral HSV reactivation, with 66.4% activity score according to the SUCRA ranking. Valacyclovir should be considered as the first line drug in patients with swallowing difficulty as well as patients with a history of poor compliance for acyclovir because of the less frequent dosing of valacyclovir compared to acyclovir [60]. Moreover, valacyclovir also offers a high level of bioavailability for cancer patients whose hospitalization solely for IV treatment is neither necessary nor desirable. This result is supported by studies by Honglund et al. [61] and by D Eisen et al. [62], where they stated that valacyclovir offers a significant advantage for bone marrow transplant patients who have difficulty taking oral drugs. Nonetheless, because of insufficient number of trials, publication bias could be identified; hence these results should be interpreted with caution. A possible explanation might be that RCTs with negative outcomes are not being published; researchers usually submit positive outcomes for publication because these results are more attractive and more likely to be considered by journals, editors and reviewed by peers. However, publication bias has an escalating and damaging impact on knowledge since it leads to significant overestimation and exaggeration of the intervention value, which will eventually lead to false conclusions that can impact the patients’ quality of life.

Our study built on existing evidence on the protective activity of acyclovir and valacyclovir against oral HSV in patients being treated for cancer. The results from the current meta-analysis can be applied practically as a guide for clinicians to choose the most effective acyclovir regimen for the prevention of oral HSV infection in patients undergoing cancer treatment. To support the findings of this network meta-analysis, we used the GRADE approach to assess the quality of evidence. According to GRADE, the quality of evidence ranged between high and moderate.

There were only two available studies that evaluated the antiviral agent’s activity in terms of oral HSV treatment in patients undergoing cancer therapy. Both studies suggested that acyclovir can significantly reduce the time needed to first reduce pain, complete pain resolution, heal and viral shedding. None of the available trials examined the effectiveness of valacyclovir for the treatment of oral HSV in patients being treated for cancer. Further trials are needed to address the questions of which antiviral agent is the most effective and safer to treat oral HSV infection in patients receiving cancer treatment and what is the optimum dose and dosage form to be used.

Several trials reported adverse effects in the acyclovir arm such as rise in hepatic transaminases Serum glutamic oxaloacetic transaminase (SGOT) and Serum glutamic pyruvic transaminase (SGPT), rash, nephrotoxicity and delirium. Similar adverse effects were reported in the placebo arm, suggesting that acyclovir is not the primary cause of these adverse effects. With respect to the elevated liver enzymes, the likelihood of acyclovir being the causative agent is very low (score D—possible rare cause of clinically apparent liver injury) and, if so, it is considered to be asymptomatic and self-limited. Liver toxicity could also be due to herpes simplex infection as hepatotoxicity and jaundice has been reported with HSV infection [63]. Moreover, it could be induced by cancer cytotoxic treatment as well [64]. There are many reasons attributed to acyclovir nephrotoxicity—for example, acyclovir is considered relatively insoluble in urine; however, approximately 60–90% of acyclovir will be excreted unchanged by the kidney which may lead to crystal-induced nephropathy. The clinical manifestations of crystal-induced nephropathy are renal function deterioration and rapid elevation of serum creatinine within the first two days of acyclovir administration [65,66]. Adair et al. [67], reported thirty cases of nephrotoxicity associated with acyclovir in patients who were devoid of renal disease prior to acyclovir administration. Thus, in order to avoid or reduce the risk of nephrotoxicity, IV acyclovir is recommended to be infused over 1 h to prevent high acyclovir concentrations accompanied by patient hydration before and during therapy [68]. In patients with deteriorated kidney function, lower doses and longer dosing intervals might be required [65].

Likewise, National Comprehensive Cancer Network (NCCN) guidelines recommend prophylactic administration of acyclovir, famciclovir or valacyclovir against oral HSV in seropositive patients who receive chemotherapy throughout the period of neutropenia for acute leukemia and for allogeneic and autologous Haematopoietic Stem Cell transplantation (HSCT), patients [69,70]. However, the recommendations have not specified the dosage recommendations. Other guideline also suggested acyclovir and valacyclovir as a grade A recommendation with level of evidence I according to the hierarchy of evidence [71]. The Infectious Diseases Working Party (AGIHO) of the German Society for Hematology and Medical Oncology (DGHO) recommended giving 400 mg oral acyclovir three or four times per day or valacyclovir 500 mg orally twice or thrice daily as prophylaxis against oral HSV [25]. The current network meta-analysis is with an agreement with all mentioned guidelines; moreover the results from this network meta-analysis results highlight the dosages which are the most effective in terms of HSV-1 prevention in patients receiving cancer treatment.

Previously, a pairwise meta-analysis by Glenny et al. [72] indicated the effectiveness of acyclovir as an intervention for the prevention and treatment of oral HSV infection. Yahav et al. [73] conducted pairwise meta-analyses to assess the antiviral prophylaxis activity against oral HSV infection and cytomegalovirus (CMV) in patients undergoing hematopoietic stem cell transplantation or intensive chemotherapy for acute leukemia or high-grade lymphoma.

HSV reactivation is very common in patients suffering from hematological malignancies and account for 37 and 68% of all oral lesions in all cancer patients [74]. The meta-analysis suggested that in the pre-engraftment setting of autologous or allogeneic (HSCT), acyclovir can significantly reduce the incidence of oral HSV infection and CMV, with no effect on overall mortality. On the other hand, in the allogeneic post-engraftment setting, acyclovir showed a significant reduction in the overall mortality, while in patients receiving chemotherapy, acyclovir showed a substantial effect in reducing oral HSV infection incidence but had no effect on mortality. Both these meta-analyses support our finding that acyclovir is an effective antiviral agent in the prevention of oral HSV infection in patients being treated for cancer.

Although conventional meta-analyses can provide a high-precision effect estimate, it addresses only one pair of comparisons, usually one intervention and placebo; consequently, it is not considered highly beneficial in case of the presence of multiple alternative interventions [75,76]. Network meta-analysis is known as a technique for comparing three or more interventions simultaneously in a single analysis by combining both direct and indirect evidence across a network of studies. Network meta-analysis provides more precise estimates than a single direct or indirect estimate [76]. By using network meta-analysis, we were able to compare the preventive activity of valacyclovir, acyclovir and placebo against oral HSV infection. Moreover, we also compared the preventive activity of different acyclovir regimens against oral HSV infection. Previous pairwise meta-analysis did not provide intervention ranking according to its effectiveness; however, we used a SUCRA curve to present an overall ranking to rank the best antiviral agent for the prevention of oral HSV infection in the first analysis, as well as the best acyclovir dose regimens in the prevention of oral HSV infection in patients being treated for cancer. Additionally, to assess the degree of confidence or certainty of our findings, we used the GRADE approach, which is the most recommended approach for the evolution of strength of evidence [77].

The current study has several limitations. Firstly, the timing of initiation of treatment was different among different trials. For instance, in the trial conducted by Saral et al. [20], 250 mg/m^2^ IV acyclovir was given either for 32 days or until the patients were discharged from the hospital or until a positive culture of herpes simplex virus infection was found. In Lundgren et al.’s study [47], 250 mg/m^2^ IV acyclovir administered twice daily was given five days before transplantation and stopped at the fifth week after transplantation. The antiviral agents’ effectiveness might be affected by these differences between protocols and may lead to analysis bias. Secondly, the included patients in different trials would have been exposed to various triggers such as sunlight and dental treatment which could have possibly triggered HSV activation. Moreover, patients had different neoplasm types and received different anticancer regimens which might influenced on HSV 1 activation, hence leading to analysis bias. The differences in genetic makeup and demographic characteristics of the subjects, trial conducts and methodology across studies can also generate considerable heterogeneity. Since only 23% of the assessed trials had an adequate allocation concealment, future studies should take into account the importance of allocation concealment in preventing selection bias and its impact on overall study results; moreover, several aspects should be encouraged in future studies such as using sensitive methods to detect oral HSV reactivation, more focused study designs and most recent protocols.

The question of which valacyclovir regimen is the most effective and safer to prevent oral HSV infection in patients receiving cancer treatment has remained controversial due to the limited number of studies, as NMA could not be performed. Encouraging RCTs to assess valacyclovir activity in the prevention of oral HSV in patients undergoing cancer treatment is highly recommended. Valacyclovir is considered more cost-effective when compared to acyclovir because valacyclovir requires less-frequent dosing and hence higher patient compliance. Dignani et al. [78] assessed the cost of prophylactic valacyclovir compared to IV acyclovir in autologous progenitor cell transplantation patients; prophylactic valacyclovir could save up to USD 60,600 for autologous progenitor cell transplantation patients.

A significant finding from our literature review is that the majority of trials examined oral HSV infection reactivation in hematological malignancies such as leukemia, myeloma and lymphoma. In contrast, few trials assessed patients with head and neck cancer and solid tumors, which suggests that patients with hematological malignancies are at a high risk of developing oral HSV infection reactivation [79]. Furthermore, the prevalence of oral HSV infection reactivation is significantly higher in the most recent studies compared to older studies; a possible explanation for this could be the more sensitive laboratory techniques that have been used lately and thereby improved detection.

Overall, our analysis results should provide guidance in choosing the right regimen tailored to an individual patient’s needs.

## 5. Conclusions

The success of cancer treatment is usually compromised by oral complications, especially HSV reactivation, which plays a part in patients’ quality of life and cancer treatment progression. This research aimed to identify the best antiviral agent to prevent or to treat oral HSV in patients being treated for cancer. Acyclovir and valacyclovir were found to be highly effective in the prevention of HSV-1 in patients being treated for cancer. Acyclovir is superior compared to valacyclovir. The best acyclovir regimens that can be prescribed by healthcare providers in order to prevent oral HSV in patients being treated for cancer are 750 mg/m^2^ IV acyclovir, 1600 mg oral acyclovir as a total daily dose and 800 mg acyclovir per day. The least effective regimen is 250 mg/m^2^ IV acyclovir. The literature review highlighted the lack of RCTs in the area of HSV-1 treatment in patients undergoing cancer treatment, as only a few studies assessed this part; hence the question of which antiviral agent is most effective in treating HSV-1 in patients undergoing cancer treatment has not been answered yet. Further RCTs are encouraged to be carried out assessing this issue. Remarkably, a small number of included articles had adequate allocation concealment; it is highly recommended to emphasize the importance of allocation concealment in reducing the risk of bias if future trials.

## Figures and Tables

**Table 1:** Characteristics of included randomized controlled trials (RCTs). Abbreviations: ACY, acyclovir; VAL, valacyclovir; PBO, placebo; O, oral; IV, intravenous.

Author (Year)	Country	Study Design	Study Comparison	Antiviral: Dose and Duration	Sample Size	Dropout Rate	Outcome: Oral HSV Infection	Duration of Treatment
Prevention of oral HSV infection								
SARAL et al., 1983 [20]	US, Maryland	randomized, double-blind, placebo controlled	ACY vs. PBO	IV ACY 250 mg/m^2^ body surface area, three times daily	30	3%	ACY: 0/15 PBO:11/15	32 days or until discharged from the hospital or until a culture-positive herpes simplex virus infection was found
Hann et al., 1983 [43]	London	randomized, double-blind	ACY vs. PBO	IV ACY, 5 mg/kg, twice daily	60	1.6%	ACY: 2/29 PBO:15/30	14 months
Bergmann et al.,1997 [53]	Denmark	randomized, double-blind, placebo controlled	ACY vs. PBO	O-ACY 400 mg, twice daily	90	0%	ACY: 1/45 PBO: 8/45	28 days
G Lundgren et al., 1985 [47]	Sweden	randomized, double-blind and placebo controlled	ACY vs. PBO	IV ACY at a dose of 250 mg/m^2^, twice daily	42	0%	ACY: 1/20 PBO: 6/22	6 months
Saral et al., 1981 [41]	US	double-blind	ACY vs. PBO	IV 250 mg/m^2^ acyclovir, every eight hours	20	0%	ACY: 0/10 PBO: 7/10	18 days
Bubley et al., 1989 [50]	US	randomized, double-blind, placebo controlled	ACY vs. PBO	O-ACY as four 200-mg capsules, twice daily	57	17.5%	ACY: 0/27 PBO: 5/30	7 weeks
Shepp et al., 1985 [48]	US	randomized, double-blind	ACY vs. PBO	IV ACY 250 mg/m^2^, once daily	30	10%	ACY: 6/14 PBO: 9/13	4 weeks
Lonnqvis et al., 1993 [52]	Sweden	double-blind	ACY vs. PBO	O-ACY 200 mg, four times daily	116	7.7%	ACY: 0/52 PBO:15/55	Duration varied
WADE et al., 1984 [46]	US	double-blind	ACY vs. PBO	O-ACY 400 mg, every four hours	49	0%	ACY: 5/24 PBO:17/25	35 days
Gluckman et al., 1983 [42]	France	double-blind	ACY vs. PBO	O-ACY 200 mg, every six hours	40	2.5%	ACY: 0/20 PBO:13/20	from day 8 to day 35 after transplant
Anderson et al., 1984 [45]	UK	double-blind	ACY vs. PBO	O-ACY 200 mg four times daily	41	2.5%	ACY: 1/20 PBO: 12/20	6 weeks
Liesveld et al., 2002 [54]	US	randomized open label	VAL vs. ACY	O-VAL 1 g, twice daily; IV-ACY 250 mg/m^2^, twice daily	30	3.3%	VAL: 0/14 ACY: 0/16	until the day of engraftment
Prentice et al., 1983 [44]	London	randomized placebo-controlled	ACY vs. PBO	IV-ACY 5 mg/kg infused over one hour, once daily	60	1.6%	ACY: 2/29 PBO:15/30	during the period of neutropenia
Shepp et al., 1987 [49]	US	double-blind	ACY vs. PBO	O-ACY 800 mg, twice daily	51	31%	ACY: 7/25 PBO:12/26	from day 31 till 75 days
Selby et al., 1989 [51]	UK	double-blind	ACY VS. PBO	IV acyclovir 5 mg kg three times daily. Children less than 12 years received 250 mg/m^2^, followed by 400 mg orally 6-hourly.	82	0%	ACY: 9/42 PBO:17/40	Over one year
Warkentin et al., 2002 [55]	Canada	single-blind, randomized, 3-group clinical trial	VAL 500 VS. VAL 250 VS. ACY 400	O-VAL (500 mg twice daily); O-VAL (250 mg twice daily); O-ACY (400 mg thrice daily)	151	0%	VAL 500 mg: 8/48 VAL 250 mg:2/52 ACY: 2/52	Duration varied
Orlowski et al. [56]	Sidney	randomized open label	VAL 500 vs. VAL 1000	Every 8 h	81	14.8%	VAL 500 mg: 0/40 VAL 1000 mg: 0/41	1979 days
Treatment of oral HSV infection								
Wade et al., 1982 [57]	US	randomized, double-blind	ACY vs. PBO	IV-ACY 750 mg/m^2^ body surface area once daily	31	0%	ACY:15/17 PBO:16/17	7 days
Shepp et al., 1985 [58]	-	randomized, double-blinded, placebo-controlled	ACY vs. PBO	O-ACY 400 mg five times daily	21	0%	-	10 days

**Table 2:** Risk of bias assessment of included RCTs in network-meta analysis.

Author	Year	Random Sequence Generation	Allocation Concealment	Blinding of Participants and Personnel	Blinding of Outcome Assessment	Incomplete Outcome Data	Selective Reporting	Overall Bias
Saral	1983	+	?	+	+	+	+	?
Hann	1983	+	?	+	+	+	?	-
Bergmann	1997	+	?	+	+	+	+	?
G Lundgren	1985	?	+	+	+	+	+	?
Bubley	1989	+	+	+	+	?	-	-
Wade	1984	?	?	+	+	+	+	-
Anderson	1984	?	?	+	+	+	-	-
Shepp	1987	?	?	+	+	-	?	-
Liesveld	2002	+	+	-	-	+	+	-
Selby	1989	+	?	+	+	?	?	-
Warkentin	2002	?	?	?	+	+	+	-
Saral	1981	+	?	+	+	+	+	?
Shepp	1985	+	?	+	+	-	?	-
Lonnqvis	1993	+	+	+	+	+	?	?
Prentice	1983	?	?	?	?	+	?	?
Gluckman	1983	?	?	+	+	+	-	-

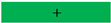
 Low risk, 
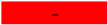
 High risk, 
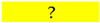
 unclear.

**Table 3:** Comparative efficacy of valacyclovir and acyclovir in the prevention of oral herpes simplex virus (HSV) infection in patients receiving cancer treatment through network meta-analysis. Abbreviations: ACY, acyclovir; VAL, valacyclovir; PBO, placebo. Note: Pairwise (upper right portion) and network (lower left portion) meta-analytic results. Outcomes are expressed as risk ratio (95% confidence intervals). For the pairwise meta-analyses, a relative risk of less than 1 indicates that the treatment specified in the row is more efficient. For the network meta-analysis, a relative risk of less than 1 shows that the treatment specified in the column is more efficient. Bold and shaded results indicate statistical significance.

ACY	**0.79** **(0.27, 2.27)**	**0.18** **(0.12, 0.27)**
0.79 (0.25, 2.48)	VAL	NA
**0.17** **(0.10, 0.30)**	**0.22** **(0.06, 0.78)**	PBO

**Table 4:** Results of network meta-analysis: Incidence of herpes simplex virus, with surface under the cumulative ranking (SUCRA) ranking. Abbreviations: ACY, acyclovir; VAL, valacyclovir; PBO, placebo.

Intervention	Incidence of Herpes Simplex Virus	
	RR (95% CI)	SUCRA Rank (Score)
ACY	0.17 (0.10, 0.30)	1 (0.83)
VAL	0.22 (0.06, 0.77)	2 (0.66)
PBO	Reference	3 (0.005)
Overall inconsistency Chi-square (*p* value)	1.87 (0.60)	
Number of studies	16	

**Table 5:** Comparative efficacy of different acyclovir doses in the prevention of oral HSV infection in patients receiving cancer treatment.

IV-ACY10MG/KG	NA	NA	NA	NA	NA	NA	**0.14** **(0.05, 0.37**)
0.33 (0.09, 1.27)	IV-ACY250MG/M2	NA	NA	NA	NA	NA	0.41 (0.17, 1.02)
0.63 (0.06, 6.13)	1.88 (0.20, 17.79)	IV-ACY500MG/M2	NA	NA	NA	NA	0.22 (0.03, 1.73)
2.48 (0.28, 21.74)	7.42 (0.87, 62.95)	3.96 (0.23, 66.89)	IV-ACY750MG/M2	NA	NA	NA	**0.06** **(0.01, 0.39)**
2.25 (0.25, 20.58)	6.72 (0.76, 59.63)	3.58 (0.20, 62.69)	0.91 (0.06, 14.53)	O-ACY1600	NA	NA	**0.06** **(0.01, 0.45)**
0.75 (0.17, 3.25)	2.25 (0.54, 9.27)	1.20 (0.12, 12.32)	0.30 (0.03, 2.80)	0.33 (0.03, 3.22)	O-ACY2000	NA	**0.18** **(0.06, 0.55)**
1.95 (0.43, 8.76)	5.82 (1.36, 25.00)	3.10 (0.29, 32.72)	0.78 (0.08, 7.44)	0.87 (0.09, 8.57)	2.59 (0.53, 12.60)	O-ACY800	**0.07** **(0.02, 0.22)**
**0.14** **(0.05, 0.37)**	0.41 (0.17, 1.02)	0.22 (0.03, 1.73)	**0.06 (0.01, 0.39)**	**0.06** **(0.01, 0.45)**	**0.18 (0.06, 0.55)**	**0.07 (0.02, 0.22)**	PBO

**Table 6:** Incidence of herpes simplex virus with different acyclovir regimens and SUCRA ranking. Abbreviations: PBO, placebo; O-ACY, oral acyclovir; IV-ACY, intravenous acyclovir.

Intervention	Incidence of Herpes Simplex Virus	
	RR (95% CI)	SUCRA Rank (Score)
IV-ACY 750MG/M2	0.06 (0.01, 0.39)	1 (0.79)
O-ACY 1600	0.06 (0.01, 0.45)	2 (0.77)
O-ACY 800	0.07 (0.02, 0.22)	3 (0.76)
IV-ACY 10MG/KG	0.14 (0.05, 0.37)	4 (0.56)
O-ACY 2000	0.18 (0.06, 0.55)	5 (0.46)
IV-ACY 500MG/M2	0.22 (0.03, 1.73)	6 (0.42)
IV-ACY 250MG/M2	0.41 (0.17, 1.02)	7 (0.22)
PBO	reference	8 (0.02)
Overall inconsistency Chi-square (*p* value)	Inconsistency model requested, but there is no source of inconsistency. No tests were performed.	
Number of studies	13	

**Table 7:** Assessment of global inconsistency for risk of incidence of oral HSV in networks using “design-by-treatment” interaction model.

Network Outcome	Chi Square	*p* Value for Test of Global Inconsistency
Incidence of oral HSV by drug class	1.87	0.60
Incidence of oral HSV by dosage form and total daily dose	-	-

**Table 8:** GRADE summary evidence for primary analysis on prevention of oral HSV infection. Abbreviation: *, Risk of bias; ^§^, Severe imprecision; ^‡‡^, Indirectness or intransitivity; ^¶^, Contributing direct evidence of moderate quality.

Comparison	Direct Evidence		Indirect Evidence		Network Meta-Analysis	
	Odds Ratio (95% Confidence Interval)	Quality of Evidence	Odds Ratio (95% Confidence Interval)	Quality of Evidence	Odds Ratio (95% Confidence Interval)	Quality of Evidence
ACY vs. PBO	0.18 (0.12, 0.28)	Moderate *^,‡‡^	0.17 (0.13, 0.24)	Moderate ^¶,‡‡^	0.17 (0.10, 0.30)	Moderate
ACY vs. VAL	0.79 (0.27, 2.27)	Moderate *	0.79 (0.01, 608.3)	Low ^¶,‡‡,§^	0.79 (0.25, 2.48)	Moderate

**Table 9:** GRADE summary evidence for the subgroup analysis (comparison of total daily dose of acyclovir and dosage forms in the prevention of oral HSV incidence). There is no triangle loop formed. Hence, it is impossible to have indirect evidence. Abbreviation: *, Risk of bias; ^‡^, imprecision; ^‡‡^, Indirectness or intransitivity; ^††^, Cannot be estimated because the drug was not connected in a loop in the evidence network.

Comparison	Direct Evidence		Indirect Evidence		Network Meta-Analysis	
	Odds Ratio (95% Confidence Interval)	Quality of Evidence	Odds Ratio (95% Confidence Interval)	Quality of Evidence	Odds Ratio (95% Confidence Interval)	Quality of Evidence
IV-ACY 10MG/KG vs. PBO	0.14 (0.05, 0.37)	High *	Not estimable ^††^	Not estimable ^††^	0.14 (0.05, 0.37)	High
IV-ACY 250MG/M2 vs. PBO	0.41 (0.17, 1.02)	Moderate *^,‡^	Not estimable ^††^	Not estimable ^††^	0.41 (0.17, 1.02)	Moderate
IV-ACY 500MG/M2 vs. PBO	0.22 (0.03, 1.73)	Moderate *^,‡^	Not estimable ^††^	Not estimable ^††^	0.22 (0.03, 1.73)	Moderate
IV-ACY 750MG/M2 vs. PBO	0.06 (0.01, 0.39)	High	Not estimable ^††^	Not estimable ^††^	0.06 (0.01, 0.39)	High
O-ACY 1600 vs. PBO	0.06 (0.01, 0.45)	Moderate *^,‡‡^	Not estimable ^††^	Not estimable ^††^	0.06 (0.01, 0.45)	Moderate
O-ACY 2000 vs. PBO	0.18 (0.06, 0.55)	High *	Not estimable ^††^	Not estimable ^††^	0.18 (0.06, 0.55)	High
O-ACY 800 vs. PBO	0.05 (0.01, 0.22)	Moderate *^,‡‡^	Not estimable ^††^	Not estimable^††^	0.07 (0.02, 0.22)	Moderate

## Appendix A

**Table A1:** Search terms used in search strategy.

#	Keyword	Medline (Ovid)	Embase	Cochrane Central
1	exp NEOPLASMS/	3,327,198	4,250,169	77,595
2	exp LEUKEMIA/	231,072	281,659	4644
3	exp LYMPHOMA/	170,934	278,418	3173
4	exp RADIOTHERAPY/	184,530	502,025	5856
5	Chemotherapy.mp.	434,962	782,593	78,184
6	Bone Marrow Transplantation/	44,512	48,482	1368
7	neoplasm$.mp.	2,803,199	756,571	79,397
8	cancer$.mp.	1,769,905	3,491,963	169,046
9	(leukaemi$ or leukemi$).mp.	326,549	437,588	15,026
10	(tumour$ or tumor$).mp.	2,085,918	2,992,357	78,405
11	malignan$.mp.	569,105	861,406	27,680
12	neutropeni$.mp.	44,306	128,428	14,301
13	carcino$.mp.	1,021,355	1,423,938	45,899
14	adenocarcinoma$.mp.	238,058	269,624	11,190
15	lymphoma$.mp.	242,728	314,058	12,414
16	(radioth$ or radiat$ or irradiat$ or radiochemo$).mp.	893,029	1,226,258	51,508
17	(bone adj marrow adj5 transplant$).mp	5,8628	85,127	4001
18	chemo$.mp.	753,479	1,307,643	92,912
19	or/1–18	5,244,394	6,656,084	271,167
20	herpes simplex/or herpes labialis/or stomatitis, herpetic/	15,890	19,232	462
21	(herpes or herpetic or “cold sore$”or cold-sore$).mp.	72,420	116,809	4872
22	simplexvirus/or herpesvirus 1, human/	27,442	3361	195
23	HSV$.mp.	25,977	32,427	862
24	fever blister$.mp.	39	53	5
25	(oral adj3 infection).ti,ab.	4631	5331	430
26	(oral and vir$).ti,ab.	27,253	36,737	4175
27	or/20–26	106,267	157,655	9211
28	exp Randomized Controlled Trial/	508,037	603,738	131
29	exp Clinical Trial/	861,035	1,486,491	163
30	randomized controlled trial.pt.	507,205	0	495,880
31	random$.tw.	1,113,139	1,526,873	956,392
32	blind$.ti,ab.	291,995	408,397	319,311
33	placebo$.ti,ab.	212,210	302,942	298,344
34	trial$.ti,ab.	1,005,908	1,432,033	652,484
35	or/28–34	2,163,766	3,165,315	1,236,237
36	exp Acyclovir/	14,207	38,744	1205
37	exp Zovirax/	14,207	38,744	1205
38	antiviral$.ti,ab.	85,534	115,959	5684
39	exp Valacyclovir/	1060	7794	-
40	exp Valtrex/	1060	7794	-
41	exp Famciclovir/	529	3865	-
42	exp Famvir/	529	3865	-
43	anti viral$.ti,ab.	6856	11,216	448
44	antiherpetic.mp.	519	638	16
45	exp Acycloguanosine/	14,207	38,744	1205
46	or/36–45	102,879	161,355	or/36–41 7036
47	19 AND 27 AND 35 AND 46	599	1863	197
48	limit 47 to humans limit 47 to (english language and humans)	484	1680	151
